# Ultra-high piezoelectric coefficients and strain-sensitive Curie temperature in hydrogen-bonded systems

**DOI:** 10.1093/nsr/nwaa203

**Published:** 2020-08-28

**Authors:** Yangyang Ren, Menghao Wu, Jun-Ming Liu

**Affiliations:** School of Physics, Huazhong University of Science and Technology, Wuhan 430074, China; School of Physics, Huazhong University of Science and Technology, Wuhan 430074, China; Laboratory of Solid State Microstructures, Nanjing University, Nanjing 210093, China

**Keywords:** hydrogen-bonded ferroelectrics, strain-sensitive Curie temperature, ultra-high piezoelectric coefficient, first-principles calculations, Monte Carlo simulations

## Abstract

We propose a new approach to obtain ultra-high piezoelectric coefficients that can be infinitely large theoretically, where ferroelectrics with strain-sensitive Curie temperature are necessary. We show the first-principles plus Monte Carlo simulation evidence that many hydrogen-bonded ferroelectrics (e.g. organic PhMDA) can be ideal candidates, which are also flexible and lead-free. Owing to the specific features of hydrogen bonding, their proton hopping barrier will drastically increase with prolonged proton transfer distance, while their hydrogen-bonded network can be easily compressed or stretched due to softness of hydrogen bonds. Their barriers as well as the Curie temperature can be approximately doubled upon a tensile strain as low as 2%. Their Curie temperature can be tuned exactly to room temperature by fixing a strain in one direction, and in another direction, an unprecedented ultra-high piezoelectric coefficient of 2058 pC/N can be obtained. This value is even underestimated and can be greatly enhanced when applying a smaller strain. Aside from sensors, they can also be utilized for converting either mechanical or thermal energies into electrical energies due to high pyroelectric coefficients.

## INTRODUCTION

Ferroelectrics with a reversible spontaneous electric polarization are also piezoelectric as their magnitude of polarizations can be tuned via applying a mechanical stress. Piezoelectric effect is closely related to the occurrence of electric dipole moments in solids, where the change of polarization P upon strain might either be caused by a reconfiguration of the dipole-inducing surrounding or by re-orientation of dipole moments under the influence of the external stress. It can convert mechanical energy into electrical energy and vice versa, which can find applications such as sensors, actuators or ultrasonic motors.

**Figure 1.**
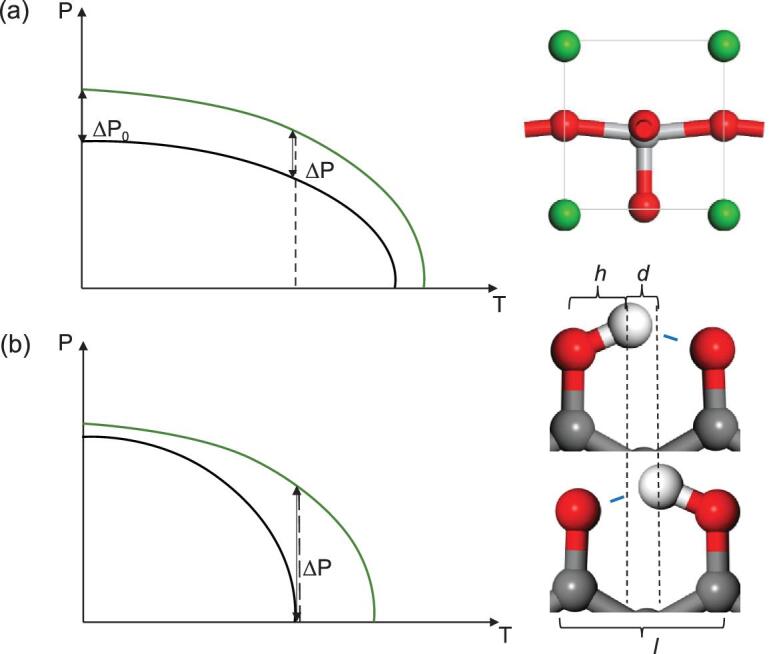
The change of polarization upon a strain in (a) perovskite ferroelectrics and (b) HP ferroelectrics, where the black/green curves represent the dependence of polarization on temperature before/after a tensile strain is applied. Red, white and gray spheres denote O, H and C atoms, respectively.

Prevalent piezoelectric materials like barium titanate (BaTiO_3_), lead titanate (PbTiO_3_) and lead zirconate titanate (PZT) as well as emerging organic–inorganic hybrid systems possess piezoelectric coefficients 20–800 pC/N [1–5], which are also ferroelectric (FE). For many ferroelectric perovskite crystals like BaTiO_3_ or PbTiO_3_, their Curie temperatures are mostly far above room temperature, so the change of polarization ΔP upon a strain at room temperature is approximately the same as ΔP_0_ at *T* = 0 K, as shown in Fig. 1(a). This change is usually small as an intrinsic property. Alternative strategies to enhance this change are usually needed. The origin of ultra-high piezoelectricity in PZT is generally attributed to the compositionally-induced structural change called morphotropic phase boundary (MPB) [1], which occurs in the region of the composition-temperature phase diagram where crystal structure changes from tetragonal to rhombohedral. Here we propose another possibility of inducing high piezoelectric coefficient, noting the rapid change of polarization when drawing near to the FE Curie temperature *T*_c_. As one of the well-known critical phenomena near *T*_c_, the polarization P can be written as:

(1) \begin{equation*}{\rm P} = \left\{ {\begin{array}{@{}*{1}{l}@{}} {\mu {{({T_c} - T)}^\delta },\,{\rm{as}}\,T < {T_c}}\\ {0,\,\quad\quad\quad\quad\,\,{\rm{as}}\, T > {T_c}} \end{array}} \right.,\end{equation*}

where *δ* < 0.5 is the critical exponent and a constant. We suppose that a small tensile strain ϵ can give rise to a linear increase in the Curie temperature by Δ*T *= kϵ, where k is the slope. It is seen that at *T *=* T*_c_, the change of polarization will be ΔP = μ Δ*T*^δ^ = μk^δ^ϵ^δ^, as shown in Fig. 1(b). Since δ – 1 < 0, the piezoelectric coefficient ΔP/ϵ = μk^δ^ϵ^δ-1^ will be theoretically infinitely large at *T* ∼ *T*_c_ when ϵ is drawing to be infinitely small. To obtain a large piezoelectric coefficient near the Curie temperature, two prerequisites are also needed: (i) the Curie temperature should be around the operating temperature (e.g. room temperature, which is just a bar for realistic application), (ii) the Curie temperature should be sensitive to strain, and then a high k will be favorable. For well-known ferroelectric perovskites like BaTiO_3_ or PZT, their Curie temperatures ranging from 400 to 800 K are far above room temperature. Usually their ferroelectric switching barriers are above 0.1 eV, and their bulk moduli are over a hundred GPa. It will be challenging to adjust their Curie temperature to room temperature upon a compressive strain. As illustrated in the sketch in Fig. 1(a) based on equation (1), the change of polarization upon a strain in high-*T*_c_ perovskite ferroelectrics at ambient conditions ΔP will be similar to the change at zero temperature ΔP_0_.

In this work, we note that hydrogen-bonded proton-transfer (HP) FE may meet those two requirements. For hydrogen bonds like O–H…O, each proton will be covalently bonded to only one side of the O atom due to the saturation of covalent bond, giving rise to robust symmetry breaking compared with the mixed ionic-covalent bonds in perovskite ferroelectrics [6,7]. Owing to its brittle nature, once the covalent bond is broken, new covalent bonds cannot be easily reformed due to the high energy barrier [8,9]. The proton-transfer ferroelectricity has been widely studied in various organic materials like croconic acid [10,11], PhMDA [12,13], [H-55DMBP][Hia] [14] and graphanol [15], or inorganic materials like alkali hydroxides [16]. Their Curie temperatures usually range from 200 to 400 K. Upon an electric field, the protons of O–H…O bonds or N–H…N bonds can cooperatively shift to the hydrogen-bonded neighbor and give rise to FE switching. Usually the bond lengths of O–H and O–H…O (marked as *h* and *l* in Fig. 1(b)) are respectively ∼1.0–1.1 Å and ∼2.4–2.7 Å, while the proton-transfer distance (marked as *d = l –* 2*h*) and barrier are respectively ∼0.3–0.5 Å and ∼0.03–0.1 eV. The O–H bond will be on the verge of breaking at the hopping transition state where the proton locates at the midpoint. Due to the brittle nature of covalent bond, if the O–H…O bonds are prolonged upon a tensile strain ϵ, the hopping barrier as well as Curie temperature may be greatly enhanced with a much larger transfer distance *d *+ *l*ϵ/2 (*l* >> *d*). Compared with Fig. 1(a), the change of polarization upon a strain for hydrogen-bonded ferroelectrics with *T*_c_ around room temperature in Fig. 1(b) will be much larger. Meanwhile their hydrogen-bonded network can be easily compressed or stretched due to softness of hydrogen bonds. Protons can flip from one side of the hydrogen bonds to the other side and change the polarization without inducing a strain, and the response time should be fast due to the small masses of protons. Through the first-principle calculations combined with Monte Carlo simulations, we will show the evidence for the ultra-high piezoelectricity in various HP ferroelectrics, which are all lead-free with unprecedented high piezoelectric coefficients.

## RESULTS AND DISCUSSION

**Figure 2.**
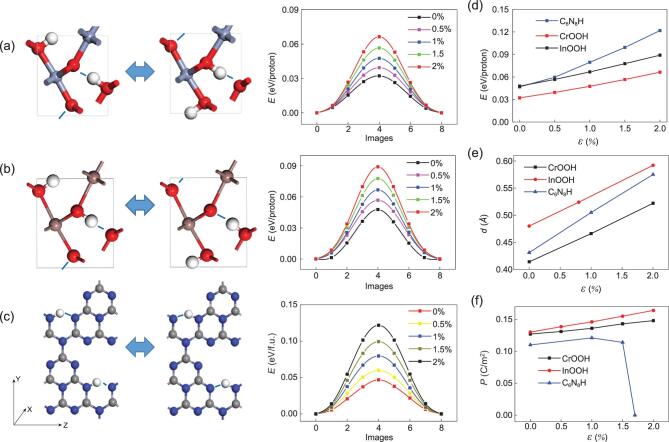
The FE switching pathway upon biaxial strains for (a) CrOOH, (b) InOOH and (c) C_6_N_8_H. Their dependences of (d) proton-transfer barrier, (e) proton-transfer distance and (f) P_0_ on strain ϵ are also displayed.

We first selected CrOOH, InOOH [17] and C_6_N_8_H [18] as three paradigmatic cases for study, which have been revealed as HP ferroelectrics/multiferroics in previous studies. Their HP FE switching pathways obtained by using nudged elastic band (NEB) method are plotted in Fig. 2(a–c), in which CrOOH possesses a switching barrier of 0.032 eV/proton, much smaller compared with 0.048 eV/proton for InOOH and 0.045 eV/proton for C_6_N_8_H. Upon a biaxial tensile strain up to 2%, their HP FE switching barriers are approximately doubled and respectively enhanced to 0.066, 0.089 and 0.122 eV. The dependences of the switching barrier on strain in Fig. 2(d) reveal a general trend of linear growth, with the slope approximately 20 to 40 meV for each per cent. The increase in switching barrier upon strain is more remarkable in C_6_N_8_H compared with InOOH and CrOOH, which accords with the comparison of the increase of their proton-transfer distances in Fig. 2(e). In comparison, the polarization P_0_ at T = 0 K is not so strain-sensitive according to its dependence on strain in Fig. 2(f), where the changes of P_0_ upon a 2% biaxial strain are both within 25% for CrOOH and InOOH.

To simulate the dependence of FE polarization on temperature, the effective Hamiltonian is necessary for the Monte Carlo simulation. The energy dependences on polarization for those systems should reveal the FE-like double-well potentials, which can be described by the Landau-Ginzburg expansion:

(2) \begin{eqnarray*}E &=& \sum\limits_i {\left( {\frac{A}{2}P_i^2 + \frac{B}{4}P_i^4 + \frac{C}{6}P_i^6} \right)}\nonumber\\ &&+ \frac{D}{2}\sum\limits_{ < i,j > } {{{\left( {{P_i} - {P_j}} \right)}^2}} ,\end{eqnarray*}

where *P_i_* is the polarization of each dipole. The first three terms describe the anharmonic double-well potential in a unit cell, and the last term represents the dipole–dipole interaction between the nearest neighboring unit cells. Owing to the anisotropy, we denote the dipole–dipole interaction between the nearest neighboring unit cells as D_a_, D_b_, D_c_ along the direction of -x, -y, -z, respectively. The structures are respectively optimized upon different strains, which gives rise to different fitting parameters. The parameters A, B C, D_a_, D_b_, D_c_ in Table S1 are obtained to fit the density functional theory (DFT) results of double-well potentials. Here the change of lattice upon strain is faster than the change of polarization [19,20], which is generally assumed to be instantaneous in Monte Carlo simulations.

**Figure 3.**
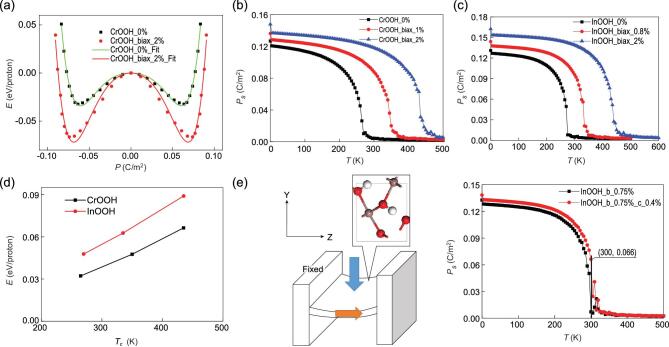
(a) Double-well potential for CrOOH with or without a strain. Temperature dependences of polarization for (b) CrOOH and (c) InOOH are plotted based on Monte Carlo simulations. (d) Dependence of switching barrier on Curie temperature. (e) A device design based on InOOH thin film where 0.75% strain is fixed along -y direction by two bar electrodes, while another strain along -z direction induced by the pressure (blue arrow) can enhance the in-plane polarization (orange arrow), which is revealed by Monte Carlo simulation.

With the effective Hamiltonian and parameters, the dependence of polarization on temperature can be obtained by the Monte Carlo simulation to investigate the Curie temperature upon different strains. Taking CrOOH as an example, the double-well potential can be well-fitted by those parameters in Fig. 3(a). The P vs. T graph is obtained by the Monte Carlo simulations based on those parameters in Fig. 3(b), where the *T*_c_ is estimated to be 265 K. Upon a biaxial strain of 1% and 2%, the *T*_c_ can be enhanced up to 350 K and 435 K, respectively. With a higher switching barrier in comparison, the estimated *T*_c_ for pristine InOOH is 270 K. Upon a biaxial strain of 0.8% and 2%, the *T*_c_ will be respectively 335 K and 435 K. Those curves can also be well-fitted by P = μ(*T*_c_ – *T*)^δ^, where δ = 0.20 for both CrOOH and InOOH.

In previous reports [21,22], mean-field theory *T*_c_ = 2Δ/3*k_B_* has been used for a rude estimation of *T*_c_ in ferromagnets, where Δ denotes the spin switching barrier. Here the dependence of FE *T*_c_ on the proton-transfer barrier is also approximately linear in Fig. 3(d), where both *T*_c_ and the barrier are approximately doubled upon a biaxial tensile strain around 2%. Although the change of polarization over strain is non-collinear, a rough estimation of average piezoelectric coefficient in certain range can be made [23,24]. A uniaxial strain along –y direction can be applied and fixed to tune the *T*_c_ to the operating temperature (e.g. ∼300 K), while a small strain along –z direction can be easily detected by the change of polarization. In the design shown in Fig. 3(e) where the strain of FE thin film along –z direction can be tuned by bending, the bar pressure marked by the blue arrow can induce a considerable change in the voltage between two bar electrodes clamping the thin film and fixing the strain in –y direction. A vertical displacement *d* for the

center of the thin film with length *l* (*d*<<*l*) along –z direction will induce a strain of 2*d*^2^/*l*^2^ along –z direction. Taking InOOH as an example, the *T*_c_ can be tuned to 300 K when a 0.75% uniaxial strain is applied along –y direction. If this strain is fixed along –y direction (denoted as ‘fixed strain’) while a small strain of 0.4% is applied along –z direction (denoted as ‘sensing strain’) at 300 K, the polarization along –z direction will increase from 0 to 6.6 μC/cm^2^ and the average piezoelectric coefficient *e*_33_ and *d*_33_ will be over 1650 μC/cm^2^ and 98 pC/N. It turns out to be higher in *e*_33_ but lower in *d*_33_ compared with PZT [1], partially owing to its large modulus *C*_33_ (∼168 GPa). At present, most research on piezoelectricity is focused on the room-temperature performance of prevalent piezoelectric materials with *T*_c_ high above room temperature, in which very few reports concern their behaviors near *T*_c_. Meanwhile there has been little attention paid to piezoelectric materials with low *T*_c_. The only case we know to date with piezoelectric behavior similar to those hydrogen-bonded systems is SbSI, also with *T*_c_ close to room temperature [25]. Previous studies have also revealed that ferromagnetism in CrOOH and C_6_N_8_H can be strengthened by a tensile strain [17,18], so those systems are also piezomagnetic, that is, ‘multipiezotronics’.

**Figure 4.**
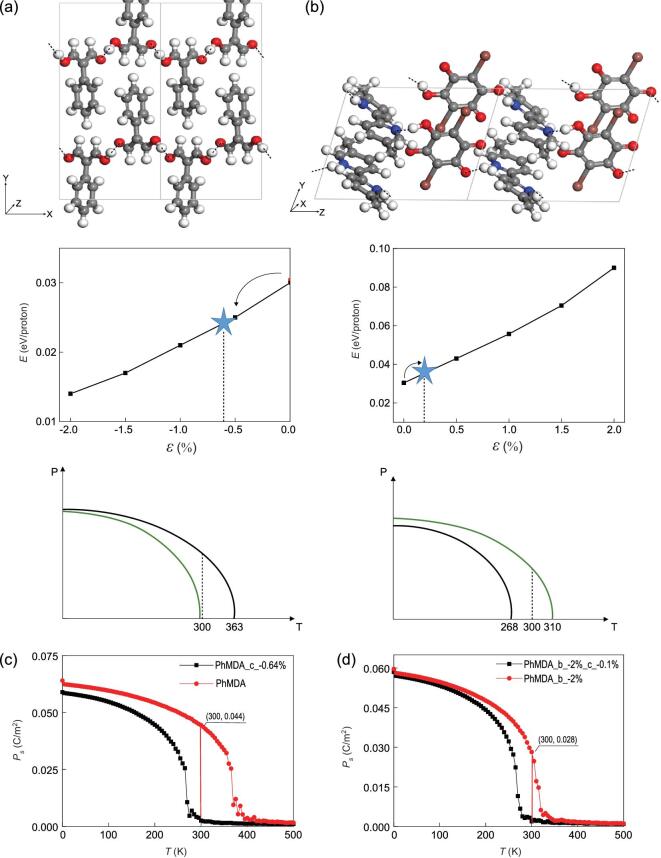
The geometric structure, dependence of proton-transfer barrier on strain, and sketches of P vs. T curves for (a) PhMDA and (b) [H-55DMBP][Hia]. Monte Carlo simulations of P–T curves of PhMDA (c) w/o a strain of −0.64% in -z direction; (d) upon a strain of −2% in -y direction w/o a strain of −0.1% in -z direction.

The systems in Fig. 2 possess relatively rigid covalent frameworks where a small tensile strain may induce large stress, while flexible piezoelectrics with soft hydrogen-bonded frameworks can be better choices. For example, a variety of organic ferroelectrics like PhMDA and [H-55DMBP][Hia] have been experimentally explored in the past decade [10,14]. Their measured Curie temperatures are respectively 363 and 268 K, which are close to room temperature and may render high peizoelectricity. According to our calculations, their polarizations at *T* = 0 K are respectively 6.5 and 5.6 μC/cm^2^ along -z direction, consistent with experimental results. Their strain sensitivity can be revealed by the dependence of proton-transfer barrier on the uniaxial strain along -z direction in Fig. 4: the barrier of PhMDA can be reduced by half upon a compressive strain less than 2%, while the barrier of [H-55DMBP][Hia] can be doubled upon a tensile strain less than 2%.

To obtain a large polarization change upon a small strain, we suppose that the Curie temperature of PhMDA changes from 363 K to 300 K upon a compressive strain, as shown in Fig. 4(a), where a rude estimation can be made first: (i) using the model P = μ(*T*_c_ − *T*)^δ^, δ = 0.20 for CrOOH/InOOH, at 300 K, ΔP/P_0_ = (363 − 300)^0.20^/363^0.20^, ΔP = 3.52 μC/cm^2^; (ii) suppose a linear relationship between *T*_c_ and Δ as in Fig. 3(d): as *T*_c_ declines from 363 K to 300 K, Δ should decrease from 0.0294 eV to 0.294 × 300/363 = 0.0243 eV, corresponding to a slight strain of −0.64% along –z direction. During the compression from 0 to −0.64%, the *e*_33_ and *d*_33_ on average would be respectively 551 μC/cm^2^ and 403 pC/N, already higher than most values measured in PZT [1]. Similarly, we suppose that the Curie temperature of [H-55DMBP][Hia] changes from 268 K to 310 K upon a tensile strain, as shown in Fig. 4(b): (i) ΔP/P_0_ = (310 − 300)^0.20^/310^0.20^, ΔP = 2.82 μC/cm^2^, (ii) as *T*_c_ increases from 268 K to 310 K, Δ should increase from 0.0286 to 0.0331 eV, corresponding to a slight strain of 0.15% along –z direction. During the extension from 0 to 0.15%, the *e*_33_ and *d*_33_ on average would be respectively 2236 μC/cm^2^ and 641 pC/N, even higher than PhMDA. Compared with InOOH, PhMDA and [H-55DMBP][Hia] possess much lower *C*_33_ ∼13.7 and 34.9 GPa, respectively, so their *d*_33_ can be greatly enhanced.

Those rough estimations can be further checked by Monte Carlo simulation based on Landau-Ginzburg model. Taking PhMDA as an example, the parameters A, B C, D_a_, D_b_, D_c_ in Table S1 are obtained to fit the DFT results of double-well potentials, and with the effective Hamiltonian and parameters, the Curie temperature is estimated to be 365 K in Fig. 4(c), very close to the experimental value of 363 K. The P-T curve can also be well-fitted by P = μ(*T*_c_ – *T*)^δ^, where δ = 0.20 is the same as CrOOH and InOOH. During the compression from 0 to −0.64% along –z direction, the *e*_33_ and *d*_33_ on average would be respectively 694 μC/cm^2^ and 507 pC/N, about 20% higher compared with the rough estimations above. We can further obtain higher piezoelectricity using the approach in Fig. 3(e), where the polarization will be more strain-sensitive in the polarization direction as a fixed strain is applied in another direction. For PhMDA, *T*_c_ will be tuned from 363 K to 315 K when a compressive strain of −2.0% is applied in the –y direction, as revealed in Fig. 4(d) by Monte Carlo simulation. If this strain is fixed while another small compressive sensing strain of −0.1% is applied along –z direction at 300 K, the polarization along –z direction will decrease from 0.0282 C/m^2^ to 0 and the average piezoelectric coefficient *e*_33_ and *d*_33_ will be respectively over 2820 μC/cm^2^ and 2058 pC/N, which is the highest value known to date [1].

**Figure 5.**
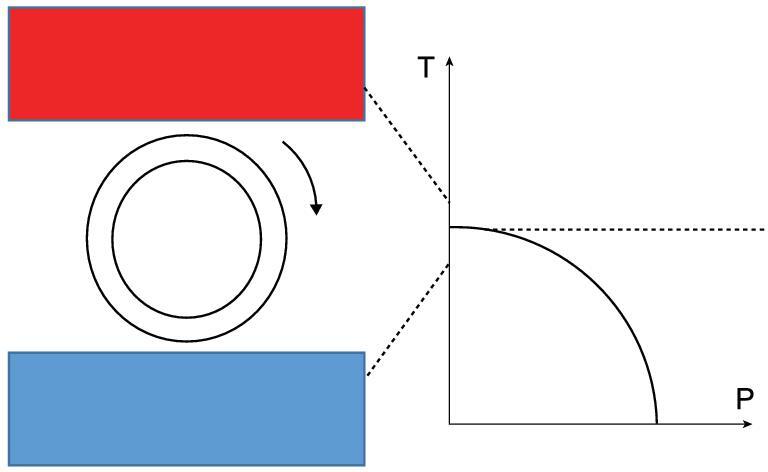
A device design of HP ferroelectrics repeatedly heated above and cooled below the Curie temperature between two thermal sources that may generate an oscillating current.

It is worth mentioning that such a high piezoelectricity only exists around *T*_c_, and ΔP/ϵ = μk^δ^ϵ^δ-1^ will decline upon increasing sensing strain ϵ. To obtain high piezoelectricity when the working temperature is altered, the fixed strain on the other direction should be tuned to adjust *T*_c_ to the new working temperature. Such tuning should be feasible due to the drastic and monotonic increase of *T*_c_ with the strain, even though *T*_c_ as well as the required fixed strain may not be accurately estimated by Monte Carlo simulations in the above cases. Those proton-transfer ferroelectrics with *T*_c_ sensitive to strain can also be utilized for energy harvesting. Even for two thermal sources with a small temperature difference (e.g. between body temperature 310 K and room temperature 300 K in Fig. 4(b)), *T*_c_ can be easily tuned to the region between two temperatures via strain. The design in Fig. 5 may give rise to an oscillating current where the HP ferroelectrics on the cover of the rotating axis are repeatedly heated and cooled, with a large pyroelectric coefficient dP/dT near the Curie temperature for efficient energy conversion. Aside from conversion of thermal energy by controlling the temperature, mechanical energy can also be converted to electrical energy using the design of nanogenerator [26,27] in Fig. 3(e).

## CONCLUSION

In summary, we propose that the Curie temperature of many hydrogen-bonded ferroelectrics can be strain-sensitive due to the unique features of hydrogen bonding, which are ideal candidates for realizing an unconventional type of ultra-high piezoelectricity. Their proton-transfer barrier as well as their Curie temperature can be approximately doubled upon a tensile strain of only 2%. For practical applications, their Curie temperature can be adjusted exactly to working-temperature by fixing a strain in one direction, and ultra-high piezoelectric coefficients (up to 2058 pC/N) that are much higher than the highest value known to date can be obtained in another direction. They can also be utilized for converting either mechanical or thermal energies into electrical energies. Since our proposed principle for such piezoelectricity can be applied to most hydrogen-bonded ferroelectrics, the large number of organic or inorganic candidates should facilitate its experimental realizations and optimizations in future, which will be a breakthrough for the long-sought lead-free high-coefficient piezoelectrics.

## METHODS

Our first-principles calculations are performed based on DFT methods implemented in the Vienna Ab initio Simulation Package (VASP 5.3.3) code [28,29]. The generalized gradient approximation in the Perdew-Burke-Ernzerhof (PBE) [30] form for the exchange and correlation potential, together with the projector-augmented-wave [31] method, are adopted. In particular, the PBE-D2 functional of Grimme [32] is used to account for the van der Waals interaction. The kinetic energy cutoff is set to be 520 eV, and computed forces on all atoms are less than 0.001 eV/Å after the geometry optimization. The Berry phase method is employed to evaluate crystalline polarization [33], and the FE switching pathway is obtained by using the NEB method [34]. A supercell of 15 × 15 × 15 unit cells was used in the Monte Carlo simulation. It has been shown by Wigner tunneling correction and also in previous studies [35,36] that a low proton-transfer barrier with short transfer distance would be further greatly reduced when the nuclear quantum effect is taken into account, while this effect is negligible in proton transfer with high barrier and long distance. This implies that the change in polarization upon a tensile strain may even be underestimated in our results neglecting the nuclear quantum effect, and the actual value of piezoelectric coefficient may be higher than our predictions.

## Supplementary Material

nwaa203_Supplemental_File
