# Supplementary material for: Ultra-high piezoelectric coefficients and strain-sensitive Curie temperature in hydrogen-bonded systems
Source: Natl Sci Rev. 2020 Aug 28;8(3):nwaa203. doi: 10.1093/nsr/nwaa203 (PMC8288374; doi:10.1093/nsr/nwaa203)
Supplement: nwaa203_Supplemental_File [file nwaa203_supplemental_file.docx]

**Supplementary Data**

Ultra-High Piezoelectric Coefficients and Strain-Sensitive Curie Temperature in Hydrogen-Bonded Systems

Yangyang Ren^1^, Menghao Wu^1^*, and Jun-Ming Liu^2^

^1^School of Physics, Huazhong University of Science and Technology, Wuhan 430074, China

^2^Laboratory of Solid State Microstructures, Nanjing University, Nanjing 210093, China

Table S1. The curie temperature T_c_(K), and the spontaneous polarization Ps(C/m^2^) at zero temperature. A(eV/(C/m^2^)^2^), B(eV/(C/m^2^)^4^) and C(eV/(C/m^2^)^6^) are used to describe the double-well potential. D_a_, D_b_ and D_c_(eV/(C/m^2^)^2^) represent the dipole-dipole interaction between the nearest neighboring dipoles along the direction of –x, -y,-z respectively.

| CrOOH | A | B | C | D_a_ | D_b_ | D_c_ | P_s_ | T_c_ |
| --- | --- | --- | --- | --- | --- | --- | --- | --- |
| 0% | -17.898 | -11068.7 | 4.38e6 | 1.28 | 3.6 | 0.74 | 0.127 | 265 |
| biax_1% | -23.99 | -12461.7 | 4.43e6 | 1.42 | 4.02 | 0.78 | 0.136 | 350 |
| biax_2% | -32.05 | -11838.2 | 3.98e6 | 1.46 | 4.7 | 0.76 | 0.148 | 435 |

| InOOH | A | B | C | D_a_ | D_b_ | D_c_ | P_s_ | T_c_ |
| --- | --- | --- | --- | --- | --- | --- | --- | --- |
| 0% | 4.09 | -39101.7 | 9.466e6 | 0.32 | 4.5 | 0.84 | 0.131 | 270 |
| biax_0.8% | 8.64 | -40659.1 | 8.2e6 | 0.308 | 4.48 | 0.86 | 0.144 | 335 |
| biax_2% | 8.6 | -37208.3 | 6.08e6 | 0.306 | 4.58 | 0.86 | 0.162 | 435 |
| b_0.75% | 4.53 | -43643.9 | 1.03e7 | 0.358 | 4.88 | 0.8 | 0.133 | 300 |
| b_0.75%_c_0.4% | 7.03 | -41930.0 | 9.1e6 | 0.332 | 4.58 | 0.786 | 0.138 | 310 |

| PhMDA | A | B | C | D_a_ | D_b_ | D_c_ | P_s_ | T_c_ |
| --- | --- | --- | --- | --- | --- | --- | --- | --- |
| 0% | 7.31 | -6.25e6 | 2.56e10 | 131.8 | 4.34 | 116 | 0.064 | 365 |
| c_-0.64% | 87.69 | -7.53e6 | 3.33e10 | 75.58 | 5.2 | 114.5 | 0.0589 | 300 |
| b_-2% | -55.75 | -5.89e6 | 2.92e10 | 100.2 | 4.76 | 127.04 | 0.0596 | 315 |
| b_-2%_c_-0.1% | -72.83 | -5.77e6 | 3.01e10 | 64.5 | 5.052 | 124.22 | 0.0585 | 300 |
